# OP53 Teledermatology — Case Study Of An Evidence Synthesis Of A Complex Intervention Using Real-World Data

**DOI:** 10.1017/S0266462325101116

**Published:** 2025-12-29

**Authors:** Joan Quigley, Orla Jenkins, Laura Rouncivell, Shibu Shrestha, Roberta Gugles, Emma Reece, Patricia Harrington, Conor Teljeur, Máirín Ryan

## Abstract

**Introduction:**

Advances in digital technologies are changing how users interact with health care. While technologies such as telehealth can be trialed in a randomized controlled trial (RCT), evidence may come predominantly from real-world data. Synthesizing this type of data is challenging, particularly for complex interventions. We present the case study of an evidence synthesis of teledermatology for triaging referrals from primary care.

**Methods:**

A systematic review was undertaken of the clinical efficacy, effectiveness, impact on service utilization, and safety of teledermatology-supported triage of primary care referrals, compared with traditional face-to-face dermatology consultations. Searching (through to June 2024), screening and extraction were undertaken as outlined in our protocol (CRD42024608084). All comparative study designs were considered, including chart reviews and single-center experiences. Risk of bias was assessed using the revised Cochrane risk-of-bias tool for randomized trials and the ROBINS-I tool for all other designs. Meta-analysis was undertaken, where appropriate, and an evidence and gap map developed.

**Results:**

Ninety-one studies were identified: 10 RCTs, two quasi-RCTs, 56 prospective non-RCTs, and 23 retrospective non-RCTs. Study samples ranged between 15 and 106,500 participants, with most completed in a single center. There was significant heterogeneity in study populations, indications, interventions, and outcome definitions and measurement. Tables, graphs, and plots (including forest plots) were used to produce a coherent narrative, assisting the decision-maker in understanding the complex evidence base. Overall findings and strength of evidence were summarized using an evidence and gap map, enabling the decision-maker to consider the balance of outcomes for teledermatology-supported triage, including clinical non-inferiority, and efficiency in management.

**Conclusions:**

As policymakers must make decisions based on the best evidence available at a point in time, it is important to capture all relevant information, including real-world data. This case study shows that real-world data can be used to inform assessments of complex interventions. Traditional evidence-synthesis presentation methods can be adapted to facilitate the inclusion of these data.

